# Effect of spatial resolution on the diagnostic performance of machine-learning radiomics model in lung adenocarcinoma: comparisons between normal- and high-spatial-resolution imaging for predicting invasiveness

**DOI:** 10.1007/s11604-025-01839-w

**Published:** 2025-07-31

**Authors:** Masahiro Yanagawa, Yukihiro Nagatani, Akinori Hata, Hiromitsu Sumikawa, Hiroshi Moriya, Shingo Iwano, Nanae Tsuchiya, Tae Iwasawa, Yoshiharu Ohno, Noriyuki Tomiyama

**Affiliations:** 1 Department of Diagnostic and Interventional Radiology, Graduate School of Medicine, The University of Osaka, 2-2 Yamadaoka, Suita-city, Osaka 565-0871 Japan; 2 Department of Radiology, Shiga University of Medical Science, Seta Tsukinowa-cho, Otsu, Shiga 520-2192 Japan; 3 Department of Radiology, Kinki-Chuo Chest Medical Center, 1180 Nagasone-cho, Kita-ku, Sakai-City, 591-8555 Japan; 4 Department of Radiology, Ohara General Hospital, 6-1 Uwamachi, Fukushima-city, Fukushima 960-861 Japan; 5 Department of Radiology, Nagoya University Graduate School of Medicine, 65 Tsurumai-cho, Showa-ku, Nagoya, 466-8550 Japan; 6 Department of Radiology, Graduate School of Medical Science, University of the Ryukyus, 207 Uehara, Nishihara-cho, Nakagami-gun, Okinawa, 903-0125 Japan; 7 Department of Radiology, Kanagawa Cardiovascular & Respiratory Center, 6-16-1 Tomioka-higashi, Kanazawa-ku, Yokohama, Kanagawa 236-0051 Japan; 8 Department of Diagnostic Radiology, Fujita Health University School of Medicine, 1-98 Dengakugakubo, Kutsukake-cho, Toyoake, Aichi 470-1192 Japan

**Keywords:** Computed tomography, High-spatial-resolution imaging, Lung cancer, Machine learning radiomics, Invasive adenocarcinoma

## Abstract

**Purpose:**

To construct two machine learning radiomics (MLR) for invasive adenocarcinoma (IVA) prediction using normal-spatial-resolution (NSR) and high-spatial-resolution (HSR) training cohorts, and to validate models (model-NSR and -HSR) in another test cohort while comparing independent radiologists’ (R1, R2) performance with and without model-HSR.

**Materials and Methods:**

In this retrospective multicenter study, all CT images were reconstructed using NSR data (512 matrix, 0.5-mm thickness) and HSR data (2048 matrix, 0.25-mm thickness). Nodules were divided into training (*n* = 61 non-IVA, *n* = 165 IVA) and test sets (*n* = 36 non-IVA, *n* = 203 IVA). Two MLR models were developed with 18 significant factors for the NSR model and 19 significant factors for the HSR model from 172 radiomics features using random forest. Area under the receiver operator characteristic curves (AUC) was analyzed using DeLong’s test in the test set. Accuracy (acc), sensitivity (sen), and specificity (spc) of R1 and R2 with and without model-HSR were compared using McNemar test.

**Results:**

437 patients (70 ± 9 years, 203 men) had 465 nodules (*n* = 368, IVA). Model-HSR AUCs were significantly higher than model-NSR in training (0.839 vs. 0.723) and test sets (0.863 vs. 0.718) (*p* < 0.05). R1’s acc (87.2%) and sen (93.1%) with model-HSR were significantly higher than without (77.0% and 79.3%) (*p* < 0.0001). R2’s acc (83.7%) and sen (86.7%) with model-HSR might be equal or higher than without (83.7% and 85.7%, respectively), but not significant (*p* > 0.50). Spc of R1 (52.8%) and R2 (66.7%) with model-HSR might be lower than without (63.9% and 72.2%, respectively), but not significant (*p* > 0.21).

**Conclusion:**

HSR-based MLR model significantly increased IVA diagnostic performance compared to NSR, supporting radiologists without compromising accuracy and sensitivity. However, this benefit came at the cost of reduced specificity, potentially increasing false positives, which may lead to unnecessary examinations or overtreatment in clinical settings.

**Supplementary Information:**

The online version contains supplementary material available at 10.1007/s11604-025-01839-w.

## Introduction

Cancer mortality rates recently tended to decline due to decreased smoking rates, advances in early detection methods for certain cancers, and improved treatment options in both adjuvant and metastatic settings [1]. However, lung cancer remains a malignant tumor with high morbidity and mortality, and its incidence continues to increase annually around the world. The predominant histological subtype of lung cancer is adenocarcinoma, with varying degrees of invasiveness classified into adenocarcinoma in situ (AIS), minimally invasive adenocarcinoma (MIA), and invasive adenocarcinoma (IVA) according to a multidisciplinary classification [2]. The degree of invasiveness significantly impacts survival rates [3]. The 5-year survival rate of AIS and MIA is nearly 100% in a completely resected state [4, 5], whereas the 5-year survival rate of IVA with pathological stage IA is 74.6% [6]. Patients diagnosed with AIS or MIA on CT may be eligible candidates for sublobar resection procedures such as segmentectomy or wedge resection [7]. Therefore, it would be of immense clinical importance to accurately predict whether it is IVA or non-IVA (AIS or MIA).

The technological advancement in spatial resolution with energy-integrating detector CT has enabled the integration of high-spatial-resolution (HSR) CT into clinical use since 2017, offering spatial resolution of up to 150 µm (in-plane) and 200 µm (through plane). Furthermore, compared to traditional CT scanners, HSR CT allows for the utilization of up to 2048 × 2048 matrix, providing exceptionally detailed information on lung anatomy and disease states [8]. It has been reported that HSR CT enhanced subjective evaluations of the pathological invasiveness in adenocarcinoma due to its improved spatial resolution [9]. However, no study so far has evaluated the software development and quantitative analysis for 2048 matrix images with 0.25 mm slice thickness. We hypothesized that the diagnostic performance of a machine learning radiomics (MLR) model using HSR CT data may surpass that of conventional CT data. This multicenter study aimed to construct two MLR models for IVA prediction using normal-spatial-resolution (NSR) and HSR training cohorts, and to validate these models (model-NSR and model-HSR) in another test cohort containing different facilities, while comparing results of two independent radiologists with and without the model-HSR.

## Materials and methods

### Study participants

**Fig. 1:**
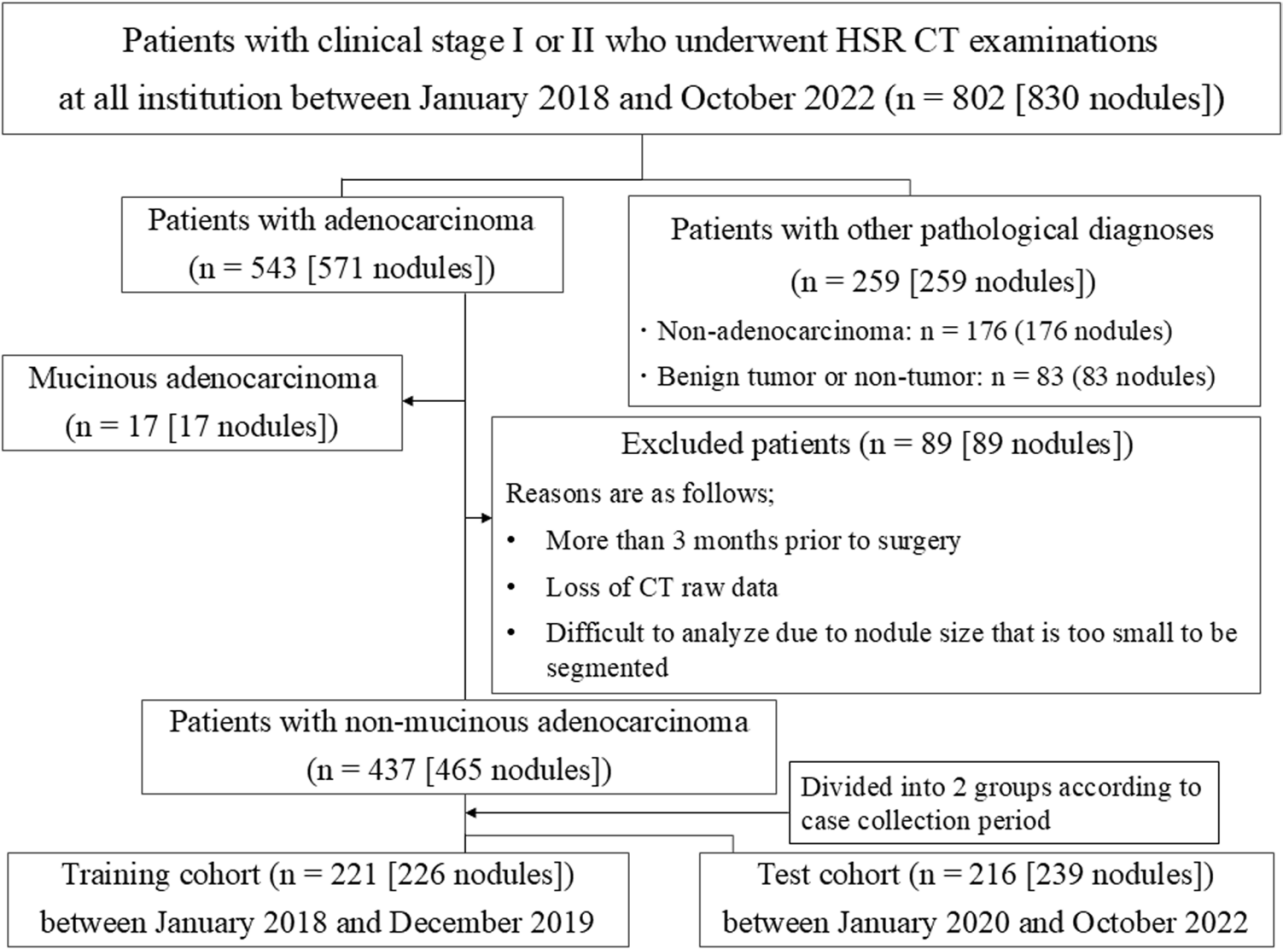
Flow chart of patient selection and demographics. *HSR* high spatial resolution. The training cohort consisted of data from six facilities, and the testing cohort consisted of data from seven facilities (including two facilities different from the six mentioned above)

This retrospective multicenter study was approved by the institutional review board of each hospital (1. Osaka University Hospital: approval number, 19,225; 2. Shiga University Hospital; approval number, R2019-288; 3. Ohara General Hospital: approval number, 195; 4. Kinki-Chuo Chest Medical Center, approval number, 2021-010; 5. University of the Ryukyus Hospital: approval number, 1559; 6. Nagoya University Hospital: approval number, 2020-0539; 7. Kanagawa Cardiovascular & Respiratory Center, approval number, KCRC-19–0040; and 8. Fujita Health University Hospital, approval number, HM20-362). Informed consent was waived for review of the patients’ records and images. The inclusion and exclusion criteria of this study was presented in Fig. 1 [Appendix E1].

### CT protocols

The super-high-resolution mode of an HSR CT scanner (Aquilion Precision; Canon Medical Systems, Otawara, Japan) equipped with a 1792-channel detector (0.25 mm × 160 rows) allowed HSR images with a 2048 × 2048 matrix and 0.25 mm slice thickness. HSR CT parameters included a helical pitch of 129, a gantry rotation period of 0.5 s, an X-ray voltage of 120 kVp, tube current regulated by auto exposure control (mA), and a field of view of 34–35 cm encompassing the full lung. All CT images were reconstructed with a standard kernel (FC13) using adaptive statistical iterative reconstruction (AIDR-3D Standard) for smoother reconstruction, which is more favorable for reproducibly extracting quantitative features [10]. When acquiring CT images with a 34 to 35 cm field-of-view, commonly used in clinical settings, the in-plane pixel size ranges from 0.166 to 0.171 mm, not exceeding the maximum spatial resolution of the CT device at 0.15 mm. The volume CT dose index (CTDIvol) was 12.4 ± 4.4 mGy, and the dose-length product was 524.7 ± 137.6 mGy-cm, obtained from the CT software. The effective dose (7.3 ± 1.9 mSv) was calculated as the product of the dose-length product and the “κ” conversion coefficient (0.014 mSv/[mGy cm]) for chest CT [11].

Normal resolution simulation (NRsim) is an algorithm that simulates NSR acquisitions using super-high-resolution raw data, generating images with accurate spatial resolution, noise, CT value accuracy, and low-contrast detectability [12]. By operating within the projection domain, the NRsim procedure avoids the need to model the numerous complex effects of linear or nonlinear reconstruction algorithms (Appendix E2).

### Histopathologic data

All histopathological specimens stained with hematoxylin–eosin and/or Elastica van Gieson staining were assessed by pathologists at each institution following the multidisciplinary adenocarcinoma criteria [2]. The final histological diagnoses (AIS, MIA, or IVA) including pleural invasion, venous invasion, and lymphatic invasion were recorded along with pathological T descriptor according to the 8th TNM classification [13].

### Outline of machine learning radiomics (MLR) software

In collaboration with Canon Medical Systems (Otawara, Japan), we developed new MLR software dedicated to HSR data (Appendix E3). A radiologic technologist, blinded to clinical and outcome information, performed 3D segmentation of each nodule for the training cohort (*n* = 221 [226 nodules]) and the test cohort (*n* = 216 [239 nodules]). The corresponding author (blinded) confirmed the accuracy of nodule extraction, excluding nodules not recognized by the software due to small size (Fig. 1).

**Fig. 2:**
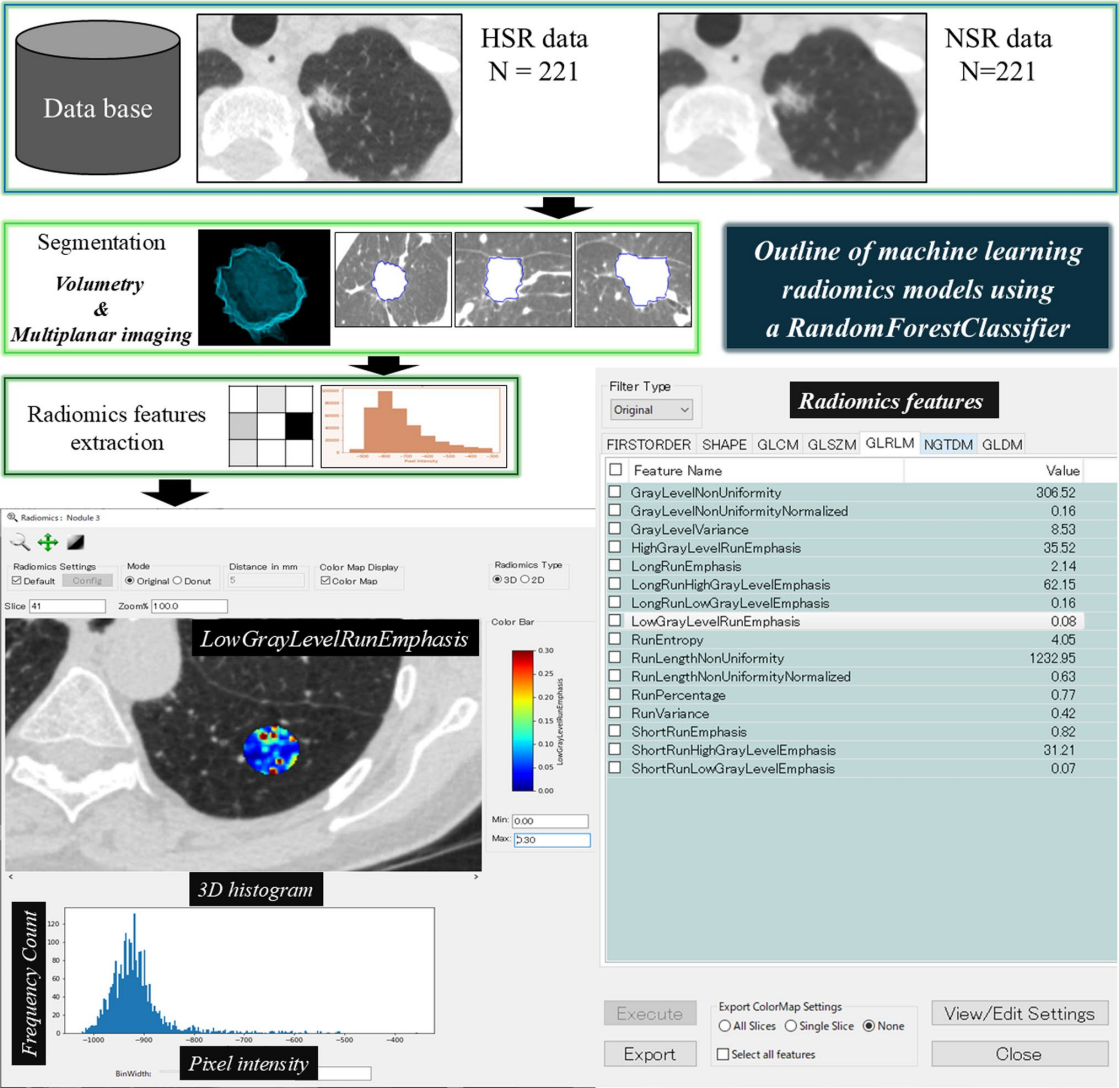
Outline of machine learning radiomics models using a RandomForestClassifier in training cohort. Nodules are automatically extracted from any CT image data, and analysis results are output using 3D CT histograms and color map displays of radiomics features. *HSR* high-spatial-resolution, *NSR* normal-spatial-resolution

The outline of MLR in the training cohort is shown in Fig. 2. To reduce explanatory variables, we applied Pearson correlation, excluding one variable from any pair with a correlation coefficient greater than 0.8 to avoid multicollinearity [14]. Additionally, explanatory variables with coefficients below 0.25 with the outcome data were excluded [14]. The correlation coefficients were calculated using [pandas.DataFrame.corr()] function (library version 2.0.3). To optimize the hyperparameters of the model, we used grid search using GridSearchCV function of the Scikit-learn library (version 1.3.0). This function comprehensively explores all possible combinations of hyperparameters defined by the user, and identifies the optimal parameter set. The hyperparameters range on RandomForestClassifier is “max_depth” {3, 5, 10}, “n_estimators” {50, 100, 200}, and “min_samples_leaf” {1, 2, 3, 5, 10}, resulting in a total of 45 parameter combinations for model. Radiomics features were selected based on feature importance values higher than 0 using the RandomForestClassifier. The model with the highest area under the curves (AUC) from receiver-operating characteristic analysis for predicting non-IVA (AIS + MIA) and IVA during cross-validation was selected as the optimal configuration. In standard k-fold cross-validation, class distributions may become uneven, especially in imbalanced datasets. In contrast, stratified k-fold cross-validation maintains the original class ratios in all training and validation set divisions. This stratification reduces bias in model evaluation and yields more robust performance. The final model performance was calculated as the simple average of 10 accuracy score in the stratified cross-validation. Both models-NSR and -HSR can create 3D CT histograms and extract analytical images displaying radiomics features with color maps.

### Evaluation of the MLR model performance

The IVA diagnostic performance of the two models (NSR- and HSR-models) was compared in a training cohort, respectively. In a test cohort containing different facilities than the training cohort, we evaluated the validity of each model by comparing between the diagnostic performance of the IVA when HSR data was input into the model-HSR and when NSR data was input into the model-NSR. We also calculated the diagnostic performance of the IVA when NSR data was input into the model-HSR.

### Subjective evaluations on HSR CT by radiologists with and without the HSR-model

In this study, all HSR CT images were reconstructed with a standard kernel (FC13) for radiomics analysis. The HSR CT images of the test cohort were evaluated independently by two chest radiologists (Y.N. and A.H.: R1 with 26 years’ experience and R2 with 15 years’ experience) using lung window settings (window width, 1500 Hounsfield units [HU]; window level, –700 HU) after processing with a high-frequency function filter. First, without the model-HSR, both radiologists diagnosed each nodule regarding the presence or absence of IVA based on HSR CT findings. The final diagnosis was determined by comprehensive evaluation of HSR CT findings for predicting IVA, including air bronchogram with disruption and/or irregular dilatation [9]. After sending the initial results to the principal investigator (M.Y. with 24 years’ experience), each radiologist independently rediagnosed the same nodules two weeks later, referencing the model-HSR results and deciding whether to change their initial assessments.

### Statistical analysis

Model building and statistical analyses were conducted using Python (v.3.8.12) and MedCalc software (version 22.014; Frank Schoonjans, Mariakerke, Belgium). The Chi-squared test was employed to identify differences in categorical classifications, including gender, lobe location, nodule type, and clinical and pathological T descriptors between AIS + MIA and IVA without multiple comparison corrections. The Mann–Whitney *U* test was used to explore differences in continuous values such as age, pack-years of smoking, and total and solid component sizes between AIS + MIA and IVA without multiple comparison corrections. For descriptive statistics in Table 1, missing values for variables (e.g., pack-years of smoking) were excluded from their respective calculations. Associations between IVA and each radiomics score were assessed using univariable logistic regression analysis, both with and without adjustments for age, sex, and pack-years of smoking. Significant parameters from the adjusted univariable analysis were included in a multiple logistic regression model, adjusted for age, sex, and pack-years of smoking (using a stepwise method with a *p* value of 0.05 for entry and > 0.1 for removal). The AUC from receiver-operating characteristic analysis was statistically analyzed using DeLong’s test to compare the predictive abilities of IVA between the two MLR models (NSR and HSR). Diagnostic performance was evaluated by comparing the accuracy, sensitivity, and specificity of two radiologists (R1, R2) with and without the HSR model using the McNemar test. A *p* value < 0.05 was considered significant.

**Table 1:** Patient characteristics

Patient Characteristic	Overall (*n* = 465)		*P* value	^a^Training cohort (*n* = 226)		*P* value	^a^Test cohort (*n* = 239)		*P* value
	AIS + MIA (*n* = 97)	IVA (*n* = 368)		AIS + MIA (*n* = 61)	IVA (*n* = 165)		AIS + MIA (*n* = 36)	IVA (*n* = 203)	
Age (year)	69 ± 10 (32–85)	70 ± 9 (34–89)	.52	67 ± 12 (32–85)	70 ± 9 (42–89)	.34	71 ± 7 (51–83)	70 ± 9 (34–86)	.66
No. of women	60 (62%)	174 (47%)	.006	35 (57%)	78 (47%)	.18	25 (69%)	96 (47%)	.004
Current or former smoker	32 (33%)	183 (50%)	.0006	21 (34%)	68 (41%)	.16	11 (31%)	115 (57%)	.003
Pack-years of smoking	10 ± 22 (0–124)	19 ± 24 (0–129)		10 ± 22 (0–124)	16 ± 25 (0–129)		11 ± 23 (0–114)	21 ± 24 (0–120)	
Lobe location			.15			.75			.07
Right upper lobe	29 (30%)	125 (34%)		17 (28%)	56 (34%)		12 (33%)	69 (34%)	
Right middle lobe	8 (8%)	27 (7%)		3 (5%)	13 (8%)		5 (14%)	14 (7%)	
Right lower lobe	19 (20%)	78 (21%)		15 (25%)	31 (18%)		4 (11%)	47 (23%)	
Left upper lobe	22 (22%)	88 (24%)		16 (26%)	39 (24%)		6 (17%)	49 (24%)	
Left lower lobe	19 (20%)	50 (14%)		10 (16%)	26 (16%)		9 (25%)	24 (12%)	
Type of nodules			< .0001			< .0001			< .0001
Ground-glass nodule	41 (42%)	19 (5%)		19 (31%)	11 (7%)		22 (61%)	8 (4%)	
Part-solid nodule	53 (55%)	214 (58%)		40 (66%)	106 (64%)		13 (36%)	108 (53%)	
Solid nodule	3 (3%)	135 (37%)		2 (3%)	48 (29%)		1 (3%)	87 (43%)	
Size of nodules (cm)									
Total diameter	1.5 ± 0.7 (0.6–4.4)	2.5 ± 0.7 (0.6–6.6)	< .0001	1.5 ± 0.7 (0.7–4.4)	2.6 ± 1.1 (0.6–6.6)	< .0001	1.4 ± 0.6 (0.6–3.0)	2.5 ± 1.1 (0.6–6.6)	< .0001
Solid component diameter	0.4 ± 0.6 (0–4.2)	1.1 ± 0.6 (0–6.6)	< .0001	0.5 ± 0.7 (0–4.2)	1.9 ± 1.2 (0–5.6)	< .0001	0.2 ± 0.4 (0–2.2)	1.9 ± 1.3 (0–6.6)	< .0001
Clinical T descriptor			< .0001			< .0001			< .0001
Tis	40 (41%)	14 (4%)		18 (29%)	6 (4%)		22 (61%)	8 (4%)	
T1mi	22 (23%)	17 (5%)		14 (23%)	4 (2%)		8 (22%)	13 (6%)	
T1a	24 (25%)	64 (17%)		19 (31%)	30 (18%)		5 (14%)	34 (17%)	
T1b	9 (9%)	134 (36%)		8 (13%)	61 (37%)		1 (3%)	73 (36%)	
T1c	1 (1%)	70 (19%)		1 (2%)	35 (21%)		0 (0%)	35 (17%)	
T2a	1(1%)	48 (13%)		1 (2%)	22 (13%)		0 (0%)	26 (13%)	
T2b	0 (0%)	14 (4%)		0 (0%)	5 (3%)		0 (0%)	9 (4%)	
T3	0 (0%)	7 (2%)		0 (0%)	2 (1%)		0 (0%)	5 (3%)	
T4	0 (0%)	0 (0%)		0 (0%)	0 (0%)		0 (0%)	0 (0%)	
Pathological T descriptor			< .0001			< .0001			< .0001
Tis	54 (56%)	0 (0%)		36 (59%)	0 (0%)		18 (50%)	0 (0%)	
T1mi	42 (43%)	1 (1%)		24 (39%)	1 (1%)		18 (50%)	0 (0%)	
T1a	1 (1%)	68 (18%)		1 (2%)	35 (21%)		0 (0%)	33 (16%)	
T1b	0 (0%)	122 (33%)		0 (0%)	53 (32%)		0 (0%)	69 (34%)	
T1c	0 (0%)	58 (16%)		0 (0%)	27 (16%)		0 (0%)	31 (15%)	
T2a	0 (0%)	84 (23%)		0 (0%)	36 (22%)		0 (0%)	48 (24%)	
T2b	0 (0%)	12 (3%)		0 (0%)	2 (1%)		0 (0%)	10 (5%)	
T3	0 (0%)	19 (5%)		0 (0%)	8 (5%)		0 (0%)	11 (5%)	
T4	0 (0%)	4 (1%)		0 (0%)	3 (2%)		0 (0%)	1 (1%)	

Age, pack-years of smoking, and size of nodules are shown as mean ± standard deviations (range). The remaining data are numbers of patients

One patient in training data and 3 patients in test data had no information about pack-years of smoking

^a^The training cohort consisted of data from six facilities, and the testing cohort consisted of data from seven facilities (including two facilities different from the six mentioned above)

*AIS* adenocarcionma in situ, *MIA* minimally invasive adenocarcinoma, *IVA* invasive adenocarcinoma

## Results

### Patients’ characteristics

Table 1 compares the characteristics of patients with AIS + MIA and IVA in the total, training cohort, and test cohorts. The training cohort consisted of data from six facilities, and the testing cohort consisted of data from seven facilities (including two facilities different from the six mentioned above). In total, 97 nodules were diagnosed as AIS + MIA and 368 nodules were diagnosed as IVA. There was no significant difference in age between AIS + MIA and IVA (*p* = 0.52). There was no significant difference in the lobe location of the tumor. The proportion of part-solid nodules and solid nodules was significantly higher in the IVA (*p* < 0.0001), and the total diameter and solid component diameter of the nodules were also significantly larger (*p* < 0.0001). Not surprisingly, there was a significant difference in the distribution of clinical and pathological T descriptors between AIS + MIA and IVA (*p* < 0.0001). In the training cohort, there was no significant difference in gender or smoking history between AIS + MIA and IVA. In all other items except for those mentioned above, both the training and test cohorts showed similar characteristics to the overall data.

### Radiomics features in NSR- and HSR-models

**Fig. 3:**
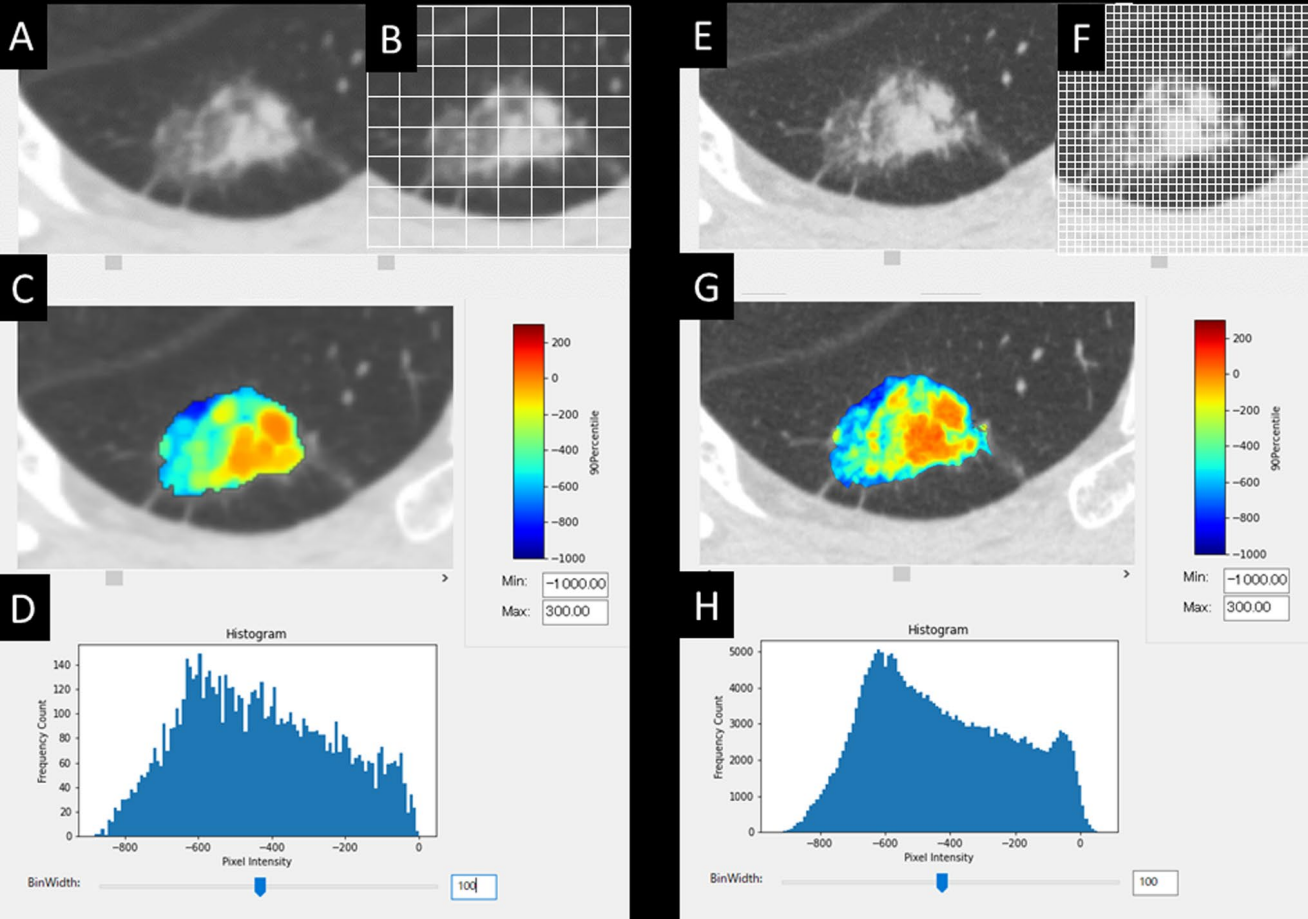
Comparisons of CT histograms and color mapping of radiomics features between NSR- and HSR-models. 71-year-old woman with a part-solid nodule measuring 1.5 cm in total diameter and 0.3 cm in solid component diameter (pathological T descriptor: pT1mi). **A** Normal-spatial-resolution (NSR) image. **B** 512 matrix. **C** Color mapping of radiomics feature from NSR image. **D** CT histograms obtained from the volumetry using NSR data. **E** High-spatial-resolution (HSR) image. **F** 2048 matrix. **G** Color mapping of radiomics feature from HSR image. **H** CT histograms obtained from the volumetry using HSR data

**Fig. 4:**
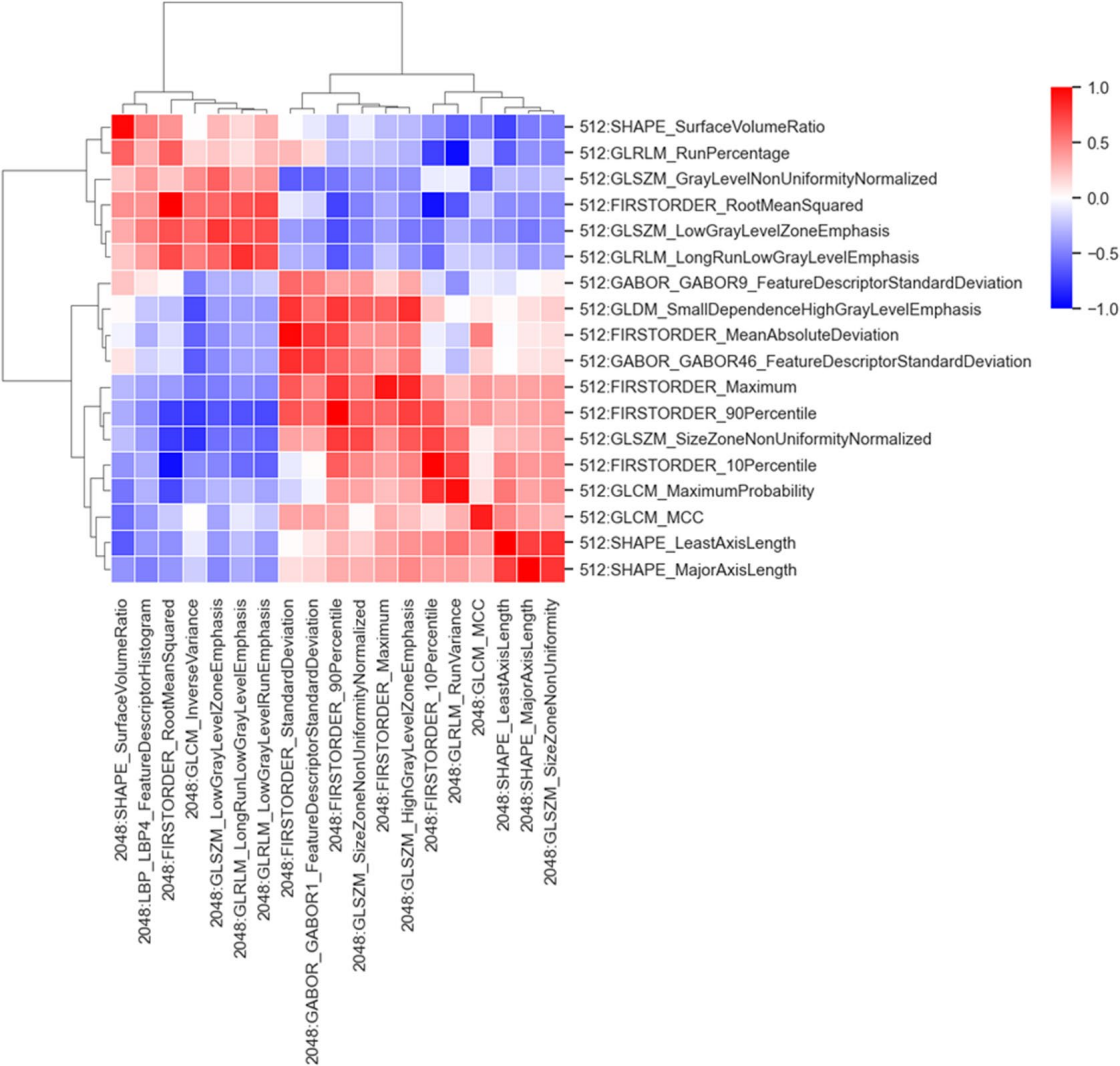
Correlations of each radiomics feature between the NSR- and HSR-models. The 18 factors in the NSR model are displayed on the vertical axis, and the 19 factors in the HSR model are displayed on the horizontal axis. Darker red indicates positive correlation, and darker blue indicates negative correlation. *HSR* high-spatial-resolution (2048 matrix), *NSR* normal-spatial-resolution (512 matrix)

CT histograms obtained from the volumetry using NSR data of 0.5-mm slice thickness and 512 matrix indicated non-smooth and irregular images, whereas those using HSR data of 0.25-mm slice thickness and 2048 matrix indicated smoother and cleaner images than NSR data (Fig. 3). In the training cohort, significant radiomics features of the RandomForestClassifier in NSR- and HSR-models are shown in Table 2. Correlations between each factor in the NSR- and HSR-models are shown in the correlation diagram (Fig. 4). In the NSR-model, 18 factors are shown in the order of feature importance by RandomForestClassifier. The top three factors are as follows: FIRSTORDER_10Percentile, FIRSTORDER_Maximum, and FIRSTORDER_RootMeanSquared. In the HSR-model, 19 factors are shown in order of feature importance by RandomForestClassifier. The top three factors are as follows: FIRSTORDER_Maximum, GLRLM_LowGrayLevelRunEmphasis, and FIRSTORDER_RootMeanSquared.

**Table 2:** Training cohort: radiomics features in normal-spatial-resolution (NSR)- and high-spatial-resolution (HSR)-models

Radiomics features in NSR-model (512 × 512 matrix size, 0.5-mm slice thickness)	Feature importance by RandomForestClassifier
FIRSTORDER_10Percentile	0.2738
FIRSTORDER_Maximum	0.1680
FIRSTORDER_RootMeanSquared	0.0783
GLCM_MCC	0.0705
FIRSTORDER_90Percentile	0.0659
GLDM_SmallDependenceHighGrayLevelEmphasis	0.0646
SHAPE_LeastAxisLength	0.0473
SHAPE_SurfaceVolumeRatio	0.0443
GLSZM_SizeZoneNonUniformityNormalized	0.0435
GLRLM_LongRunLowGrayLevelEmphasis	0.0429
GLSZM_LowGrayLevelZoneEmphasis	0.0415
GLSZM_GrayLevelNonUniformityNormalized	0.0247
GABOR_GABOR46_FeatureDescriptorStandardDeviation	0.0126
GLCM_MaximumProbability	0.0124
GABOR_GABOR9_FeatureDescriptorStandardDeviation	0.0096
FIRSTORDER_MeanAbsoluteDeviation	0.0000
SHAPE_MajorAxisLength	0.0000
GLRLM_RunPercentage	0.0000

Radiomics features in HSR-model (2048 × 2048 matrix size, 0.25-mm slice thickness)	Feature importance by RandomForestClassifier
FIRSTORDER_Maximum	0.1361
GLRLM_LowGrayLevelRunEmphasis	0.1069
FIRSTORDER_RootMeanSquared	0.1047
FIRSTORDER_90Percentile	0.1020
GLSZM_HighGrayLevelZoneEmphasis	0.0726
FIRSTORDER_10Percentile	0.0638
GLSZM_SizeZoneNonUniformity	0.0609
GLRLM_LongRunLowGrayLevelEmphasis	0.0605
GLSZM_LowGrayLevelZoneEmphasis	0.0598
GABOR_GABOR1_FeatureDescriptorStandardDeviation	0.0375
GLCM_InverseVariance	0.0356
GLSZM_SizeZoneNonUniformityNormalized	0.0300
FIRSTORDER_StandardDeviation	0.0256
SHAPE_MajorAxisLength	0.0251
LBP_LBP4_FeatureDescriptorHisogram	0.0223
SHAPE_SurfaceVolumeRatio	0.0206
SHAPE_LeastAxisLength	0.0177
GLRLM_RunVariance	0.0017
GLCM_MCC	0.0011

### Diagnostic performance of the two MLR models (NSR-model vs. HSR-model)

**Fig. 5:**
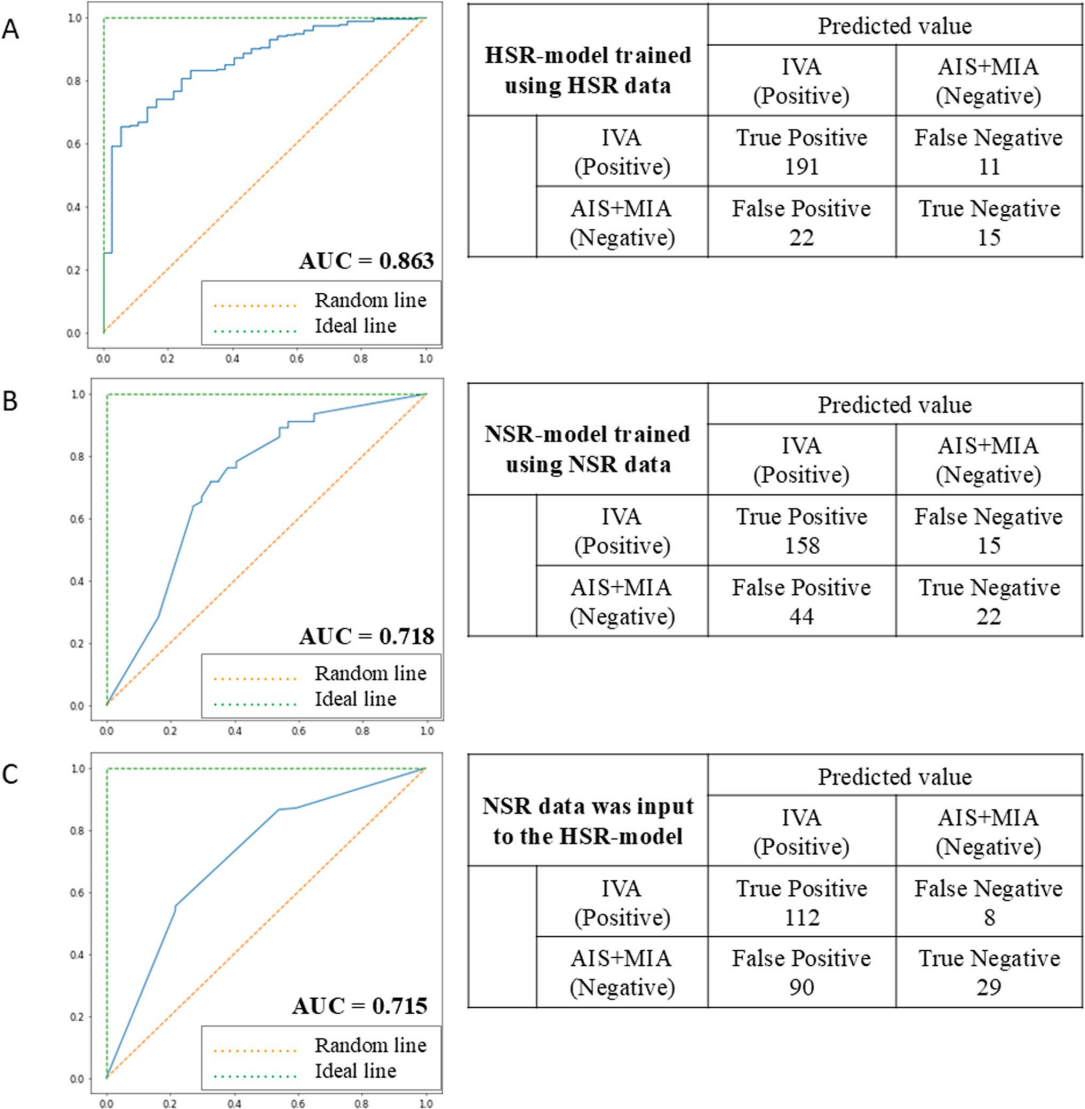
Diagnostic Performance between the NSR- and HSR**-**models. **A** Diagnostic performance of HSR-model trained using HSR data. **B** Diagnostic performance of NSR-model trained using NSR data. **C** Diagnostic performance of HSR model with NSR data. *AIS* adenocarcinoma in situ, *AUC* area under the curve, *HSR* high-spatial-resolution, *IVA* invasive adenocarcinoma, *MIA* minimally invasive adenocarcinoma, *NSR* normal-spatial-resolution

In the training cohort, the AUC of the HSR-model (0.839, 95% confidence interval [CI]: 0.756–0.922) was significantly higher than that of the NSR-model (0.723, 95% CI 0.614–0.832) (*p* < 0.01). In the test cohort, the AUC of the HSR-model (0.863, 95% CI 0.80–0.93) was also significantly higher than that of the NSR-model (0.718, 95% CI 0.62–0.82) (*p* = 0.002). Thus, the HSR-model showed significantly higher diagnostic performance than the NSR-model. However, when NSR data was input to the HSR-model trained using HSR data, the AUC (0.715, 95% CI 0.63–0.80) was slightly lower than that of the NSR model trained using NSR data (Fig. 5).

### Relationship of radiomic features with IVA in two MLR models (NSR-model vs. HSR-model)

Table 3 summarizes the results of the association of the top three radiomics features in each model with IVA in the test cohort. 236 of 239 in the test cohort was analyzed because 3 patients had no pack-years information. In the NSR-model, univariable logistic regression analyses revealed that all three radiomics features were of significant use for predicting IVA. Multivariable logistic regression analysis revealed that two radiomics features of FIRSTORDER_10Percentile (adjusted odds ratio [OR], 4.3 [95% CI 1.6, 11.4]; *p* = 0.003) and FIRSTORDER_Maximum (adjusted OR, 4.9 [95% CI 1.9, 13.1; *p* = 0.001) were indicators of IVA.

**Table 3:** Test cohort: relationship of radiomics features with invasive adenocarcinoma (IVA) in normal-spatial-resolution (NSR)- and high-spatial-resolution (HSR)-models

Radiomics features	Univariable Analysis				Univariable analysis adjusted for age, sex, and pack-years of smoking		Multivariable analysis adjusted for age, sex, and pack-years of smoking by stepwise method	
	Number of AIS + MIA	Number of IVA	OR (95% CI)	*p* value	OR (95% CI)	*p* value	OR (95% CI)	*p* value
NSR-model^a^								
FIRSTORDER_10Percentile								
Negative (*n* = 55)	22	33	8.6 (3.9, 18.8)	< .0001	9.6 (4.2, 22.2)	< .0001	4.3 (1.6, 11.4)	.003
Positive (*n* = 181)	13	168						
FIRSTORDER_Maximum								
Negative (*n* = 56)	24	32	11.5 (5.1, 25.8)	< .0001	10.1 (4.4, 23.2)	< .0001	4.9 (1.9 13.1)	.001
Positive (*n* = 180)	11	169						
FIRSTORDER_RootMeanSquared								
Negative (*n* = 95)	30	65	12.6 (4.7, 33.8)	< .0001	11.2 (4.1, 30.7)	< .0001	ND	
Positive (*n* = 141)	5	136						

HSR-model^a^	Number of AIS + MIA	Number of IVA	OR (95% CI)	*p* value	OR (95% CI)	*p* value	OR (95% CI)	*p* value
FIRSTORDER_Maximum								
Negative (*n* = 56)	24	32	11.5 (5.1, 25.8)	< .0001	9.8 (4.3, 22.8)	< .0001	4.3 (1.6, 11.3)	.004
Positive (*n* = 180)	11	169						
GLRLM_LowGrayLevelRunEmphasis								
Negative (*n* = 131)	33	98	17.3 (4.1, 74.2)	< .0001	14.9 (3.4, 64.8)	< .0001	ND	
Positive (*n* = 105)	2	103						
FIRSTORDER_RootMeanSquared								
Negative (*n* = 95)	30	65	12.6 (4.7, 33.8)	< .0001	10.8 (3.9, 29.6)	< .0001	5.0 (1.5, 16.2)	.007
Positive (*n* = 141)	5	136						

*OR* odds ratio, *CI* confidence interval, *AIS* adenocarcionma in situ, *MIA* minimally invasive adenocarcinoma, *IVA* invasive adenocarcinoma

^a^236 of 239 in the test cohort was analyzed because 3 patients had no pack-years information

In the HSR-model, univariable logistic regression analyses revealed that all three radiomics features were of significant use for predicting IVA. Multivariable logistic regression analysis revealed that two radiomics features of FIRSTORDER_Maximum (adjusted odds ratio [OR], 4.3 [95% confidence interval {CI} 1.6, 11.3]; *p* = 0.004) and FIRSTORDER_RootMeanSquared (adjusted OR, 5.0 [95% CI 1.5, 16.2; *p* = 0.007) were indicators of IVA.

### Performance for Radiologists with and without the HSR-model

Table 4 summarizes the diagnostic performance of two radiologists with and without the HSR-model. Without the HSR-model, accuracy, sensitivity, and specificity of the radiologists were as follows: R1, [77.0% (184/239), 95% CI 0.687–0.852]. [79.3% (161/203), 95% CI 0.714–0.872], and [63.9% (23/36). 95% CI 0.545–0.733]; and R2, [83.7% (200/239), 95% CI 0.764–0.909], [85.7% (174/203), 95% CI 0.789–0.926], and [72.2% (26/36), 95% CI 0.634–0.810]. With the HSR-model, accuracy, sensitivity, and specificity of the radiologists were as follows: R1, [87.2% (208/239), 95% CI 0.804–0.936], [93.1% (189/203), 95% CI 0.881–0.981], and [52.8% (19/36), 95% CI 0.430–0.626]; and R2, [83.7% (200/239), 95% CI 0.764–0.909], [86.7% (176/203), 95% CI 0.800–0.934], and [66.7% (24/36), 95% CI 0.575–0.759]. Accuracy and sensitivity of R1 was significantly higher with than without the HSR-model (*p* < 0.0001). Accuracy and sensitivity of R2 might be equal or higher with than without the model-HR, but not significant (*p* > 0.50). Specificity of R1 and R2 tended to decrease with the HSR-model, but not significant (*p* > 0.21).

**Table 4:** Performance for Radiologists with and without the high-spatial-resolution (HSR)-model

Radiologist-1 (R1) with 26 years’ experience		
Without the HSR-model	Histological diagnosis	
	IVA (positive)	AIS + MIA (negative)
IVA (positive)	161	13
AIS + MIA (negative)	42	23
With the HSR-model	Histological diagnosis	
	IVA (positive)	AIS + MIA (negative)
IVA (positive)	189	17
AIS + MIA (negative)	14	19
Radiologist-2 (R2) with 15 years’ experience		
Without the HSR-model	Histological diagnosis	
	IVA (positive)	AIS + MIA (negative)
IVA (positive)	174	10
AIS + MIA (negative)	29	26
With the HSR-model	Histological diagnosis	
	IVA (positive)	AIS + MIA (negative)
IVA (positive)	176	12
AIS + MIA (negative)	27	24

*AIS* adenocarcionma in situ, *MIA* minimally invasive adenocarcinoma, *IVA* invasive adenocarcinoma

## Discussion

This retrospective study using the HSR CT scanner with 0.25 mm slice thickness and 2048 matrix shows that MLR model with HSR data significantly increased the diagnostic performance of invasive adenocarcinoma than NSR data. In the HSR-model, univariable logistic regression analyses revealed that all three radiomics features were of significant use for predicting IVA. Multivariable analyses identified two features (FIRSTORDER_Maximum and FIRSTORDER_RootMeanSquared) as significant predictors of IVA, with adjusted ORs of 4.3 and 5.0, respectively. The HSR-model supported radiologists without compromising accuracy and sensitivity.

High-resolution imaging is crucial for lung evaluation with CT [15, 16]. HSR CT provides superior image quality over conventional CT by enhancing spatial resolution (0.15 mm in-plane, 0.20 mm through-plane) and reducing undersampling artifacts [8]. Matrix size also affects spatial resolution, especially when the pixel size exceeds the maximum resolution of the scanner [17, 18]. In this study, using a 34–35 cm field of view (FOV), the pixel size in the 2048 matrix ranged from 0.166 to 0.171 mm, which was within the maximum resolution of the HSR CT. The 2048 matrix size improved lung cancer assessment by increasing spatial resolution. Quantitative methods can vary significantly due to technical factors, and differences between CT scanners can affect texture characteristics [19–21]. The superior performance of the MLR model with HSR data in diagnosing invasive adenocarcinoma compared to NSR data was due to the high spatial resolution of HSR-CT, which allows for smooth 3D histograms and detailed radiomics feature maps. However, caution should be taken with input data for HSR models. Using NSR data in an HSR-trained model resulted in a slightly lower AUC (0.715, 95% CI 0.63–0.80) compared to the NSR-trained model (0.718, 95% CI 0.62–0.82). Ensuring that the feature distributions between the training and test datasets are comparable is critical to reflect meaningful patterns in both datasets [14]. This slight performance degradation is intriguing. We hypothesize that the HSR model struggles when these high-resolution features are attenuated or absent in NSR data because it is highly specialized in leveraging fine-grained spatial and textural information unique to HSR images. The mismatch in radiomics feature distributions and the potential misinterpretation of coarser NSR noise patterns likely caused the performance drop. Further research is needed to fully clarify this phenomenon and ensure robust cross-resolution inference, including detailed comparative analyses of feature distributions across resolutions and domain adaptation techniques.

Although radiomics is primarily data-driven, understanding the biological significance of radiomic signatures is critical for broader acceptance [22]. First, the significant radiomics feature common to both NSR and HSR models was FIRSTORDER_Maximum, a first-order feature representing the maximum CT value within the volume of interest (VOI). This feature is relatively easy to interpret as it reflects the association between solid components on CT and pathological invasiveness [23–25]. Second, FIRSTORDER_10Percentile, which was important in the NSR model, indicates that 10% of the ROI pixels fall below this value; higher values indicate nodules with high CT values overall, similar to the clinical implication of FIRSTORDER_Maximum. Third, in the HSR model, FIRSTORDER_RootMeanSquared, a measure of data variability, is important due to the histologic diversity of adenocarcinoma (i.e., acinar, papillary, micropapillary, and/or solid), reflecting tumor heterogeneity [2, 26, 27]. Finally, GLRLM_LowGrayLevelRunEmphasis, which was only significant in the univariate analysis of HSR model, highlights areas of low pixel value frequency. In nodules with low CT values such as GGNs, this feature increases with fine and granular structures, which was in the association that heterogeneous GGN was likely to be IVA [23–25]. The small number of GGNs with IVA may explain why it was not significant. Higher resolution affects 3D texture features, and FIRSTORDER_Maximum was particularly intuitive and consistent with previous interpretations. This ease of understanding increases the generalizability of the MLR model. Further studies are needed to build a more robust model.

MLR models can provide valuable insight into invasiveness and potentially improve the diagnostic performance of radiologists [28, 29]. This study showed that performance improves with HSR CT image data using a 2048 matrix. The model increased accuracy and sensitivity in diagnosing IVA but reduced specificity when used by radiologists. Yanagawa et al. [9] showed that unique HSR CT findings had the best performance for predicting the invasiveness of lung adenocarcinoma. However, some radiological features are undetectable by the human eye. Combining AI model evaluations with human assessments can address this issue and improve lung cancer diagnosis, although further research is needed. Recently, photon-counting detector CT has emerged as a major advancement, offering better detection of small nodules and airways, particularly with high spatial resolution, large matrix size, and thin slices [30–34]. Our findings will contribute to AI applications based on photon-counting CT, which is expected to be widely used in the future.

Furthermore, our study revealed interesting differences in how AI assistance impacted the two radiologists. Radiologist 1 (R1), with 26 years of experience, showed statistically significant improvements in accuracy and sensitivity with model assistance. However, Radiologist 2 (R2), with 15 years of experience and higher baseline performance, did not exhibit such significant improvements. This observation may suggest the presence of a ‘ceiling effect,’ where radiologists with already high baseline diagnostic abilities, like R2, have limited potential for improvement with AI assistance. This phenomenon is critical because it implies that the utility and impact of AI tools might vary depending on the inherent expertise level of the individual physician. Beyond experience, other factors likely contribute to such inter-reader variability in AI assistance effectiveness. These factors could include individual differences in receptivity to and trust in AI systems, varying confidence levels during their initial diagnosis, or even subtle differences in their diagnostic approaches to the task. For instance, an experienced radiologist like R1 might be more inclined to critically review cases where the AI model contradicts their initial assessment, leading to a higher rate of correction and improvement. Conversely, a radiologist like R2, with already robust performance, might rely on their established diagnostic patterns, or perhaps perceive less direct added value from the AI guidance for cases they already handle well. While our study’s design does not allow for a quantitative assessment of these specific psychological or methodological factors, the observed differential impact of AI assistance highlights their potential importance. This underscores a vital implication for the clinical introduction of AI tools: their effectiveness is not uniform and can be highly dependent on the characteristics and circumstances of the end-user. Future research should delve deeper into these human-AI interaction dynamics to optimize the integration of AI in clinical workflows.

Our study has several limitations. First, the evaluations were conducted using a multi-detector CT with a 0.25-mm section thickness and a 2048 matrix from a single manufacturer, and no comparable device is currently available for clinical use. As PCD-CT, capable of using a 1024 matrix, becomes more widespread, our results will provide baseline data for future studies. Second, in this study, we employed NSR image data generated using NRsim for direct comparison with HSR image data. While NRsim offers an ideal method to control for confounding factors by simulating NSR images from the same super-high-resolution raw data, it is important to acknowledge that it is a simulation and not a true, independently acquired NSR image. Although the fundamental validity of NRsim has been demonstrated in terms of various image quality metrics [12], subtle differences in specific image characteristics, such as noise texture, fine texture patterns, and the appearance of certain artifacts, might exist between NRsim-generated images and those acquired directly under native NSR conditions. These minute differences, which are not perfectly replicated by any simulation, could potentially influence the extraction of highly granular radiomics features. For instance, subtle variations in noise properties might impact higher-order texture features. While we believe NRsim remains the most appropriate method for this comparative study given the practical impossibility of acquiring identical true NSR and HSR scans, we recognize that these potential subtle discrepancies could theoretically introduce an unknown bias. This bias might lead to either an underestimation of the true performance of a native NSR model or, conversely, an overestimation of the superiority of the HSR model, particularly in the context of radiomics feature analysis. Future studies aiming to validate the clinical applicability of HSR radiomics could consider incorporating datasets where both native NSR and HSR acquisitions are available, though this presents significant logistical challenges. Third, segmentation of nodules, including surrounding ground-glass opacity, was performed by a single radiologic technologist using our custom software, with tracings reviewed by the corresponding author. While we aimed for high accuracy, a quantitative evaluation of segmentation reproducibility was not performed. This is a limitation because radiomics features are sensitive to precise delineation of the volume of interest. The absence of such quantitative reproducibility data may limit the generalizability and robustness of the extracted features and, consequently, the overall model. Fourth, the study data were from surgical cases for histological diagnoses, and the high proportion of IVA may have influenced the model output. This applies to both the training and test cohorts, so it is unlikely to have affected model performance. However, imbalance for invasiveness of nodules would have affected sensitivity and specificity estimates from radiologists. Our results of radiologists may reflect a shift in the diagnostic tendency of radiologists to overdiagnose nodules as IVA when presented with radiomic information that emphasizes invasiveness. While this may raise concerns about overtreatment, we believe it is critical to balance this potential limitation with the improved sensitivity and overall accuracy. Improved sensitivity ensures fewer false negatives, reducing the risk of missing IVA which could lead to delayed treatment. Further study is needed using a larger cohort to ensure the robustness of our findings. Finally, while the MLR model predicts IVA and non-IVA (AIS or MIA) from a prognostic perspective, actual prognosis was not analyzed. Future studies should investigate the impact of HSR CT data on models that directly predict prognosis.

In conclusion, MLR model trained by HSR data can greatly enhance diagnostic performance of invasive adenocarcinoma, providing support to radiologists without compromising accuracy and sensitivity. However, this benefit came at the cost of reduced specificity, potentially increasing false positives, which may lead to unnecessary examinations or overtreatment in clinical settings.

## Supplementary Information

Below is the link to the electronic supplementary material.

Supplementary file1 (DOCX 48 KB)
