# Supplementary material for: Effect of spatial resolution on the diagnostic performance of machine-learning radiomics model in lung adenocarcinoma: comparisons between normal- and high-spatial-resolution imaging for predicting invasiveness
Source: Jpn J Radiol. 2025 Jul 31;43(12):2003–17. doi: 10.1007/s11604-025-01839-w (PMC12647350; doi:10.1007/s11604-025-01839-w)
Supplement: Supplementary file 1 — Supplementary file1 (DOCX 48 KB) [file 11604_2025_1839_MOESM1_ESM.docx]

**Supplemental Materials**

**Appendix E1:**

**Study Participants**

A search by a principal investigator (M.Y.) and investigators at each institution (1. Osaka University Hospital: approval number, 19225; 2. Shiga University Hospital; approval number, R2019-288; 3. Ohara General Hospital: approval number, 195; 4. Kinki-Chuo Chest Medical Center, approval number, 2021-010; 5. University of the Ryukyus Hospital: approval number, 1559; 6. Nagoya University Hospital: approval number, 2020-0539; 7. Kanagawa Cardiovascular & Respiratory Center, approval number, KCRC-19-0040; and 8. Fujita Health University Hospital, approval number, HM20-362) of patients who underwent surgery for lung cancer at our institution between January 2018 and October 2022 identified 802 individuals with preoperative HSR CT. The corresponding author had complete control over the data and the information submitted for publication. Of these, a total of 802 patients with 830 nodules HSR CT were considered for inclusion. The inclusion criteria were as follows: *(a)* CT examination within 3 months prior to surgery, *(b)* operable patients with clinical stage I or II, *(c)* no previous treatment in the lungs, *(d)* age 20 years or older, and *(e)* non-mucinous adenocarcinoma.

Of the 830 nodules, 571 nodules were histopathologically confirmed as adenocarcinoma. Out of the 554 nodules identified in 526 patients with non-mucinous adenocarcinoma, additional exclusions were made for patients whose CT scans were conducted more than 3 months before surgery, those for whom image reconstruction was not possible due to lack of raw CT data, and those with nodules deemed difficult to analyze due to nodule size that is too small to be segmented. Finally, 465 nodules identified in 437 patients were divided into 2 groups according to case collection period: training cohort (n = 221 [226 nodules]) between January 2018 and December 2019, and test cohort (n = 216 [239 nodules]) between January 2020 and October 2022.

**Appendix E2:**

**Normal resolution simulation (NRsim)**

Initially, the projection data obtained in super-high-resolution modes undergo conversion from post-log raw data into the format of a detector signal value. Subsequently, these data are amalgamated across detector elements to downsample from 1792 channels to the 896 channels characteristic of the normal-resolution mode in the mid-plane and, for super-high-resolution mode, from 160 rows to 80 rows in the longitudinal direction. These downsampled raw data are then reconverted into post-log projection data and reconstructed through one of the standard reconstruction processes tailored for NSR data. In this study, NSR data (512 × 512 matrix size, 0.5-mm slice thickness) and HSR data (2048 × 2048 matrix size, 0.25-mm slice thickness) were reconstructed from raw CT data.

**Appendix E3:**

**Dedicated Machine-learning Radiomics Software for HSR data**

In collaboration with Canon Medical Systems, we developed a new machine-learning radiomics software dedicated for high-spatial-resolution (HSR) CT. Patient DICOM image were loaded to an in-house software program (Radiomics Texture Analysis). This in-house software program is based on Pyradiomics which is open-source package for extracting radiomics features, and it has a graphical user interface written by Python (v.3.8.12) which support 512, 1024 and 2048 matrix image for texture analysis. The 3D segmentation of each nodule was automatically performed for CT data. The corresponding author (*M.Y.*) visually confirmed that the nodule extraction area was accurate. Total 172 radiomics features were calculated and exported (Table S1): First-order (n=19), Shape (n=25), Gray Level Co-occurence Matrix (GLCM) (n=24), Gray Level Size Zone Matrix (GLSZM) (n=16), Gray Level Run Length Matrix (GLRLM) (n=16), Neighbouring Gray Tone Difference Matrix (NGTDM) (n=5), Gray Level Dependece Matrix (GLDM) (n=14), Gabor (n=43), and local binary pattern (LBP) (n=10) [1-10].

**References**

1. Lorensen, William & Cline, Harvey. Marching Cubes: A High Resolution 3D Surface Construction Algorithm. ACM SIGGRAPH Computer Graphics. 1987;21:163-. 10.1145/37401.37422.

2. Haralick, Robert & Shanmugam, Kalaivani & Dinstein, Ih. Textural Features for Image Classification. IEEE Trans Syst Man Cybern. 1973; SMC-3:610-621.

3. Thibault, Guillaume & FERTIL, Bernard & Navarro, Claire & Pereira, Sandrine & Lévy, Nicolas & Sequeira, Jean & Mari, Jean-Luc. 2009. Texture Indexes and Gray Level Size Zone Matrix Application to Cell Nuclei Classification. 10th International Conference on Pattern Recognition and Information Processing.

4. M. Amadasun and R. King, "Textural features corresponding to textural properties," in IEEE Transactions on Systems, Man, and Cybernetics, vol. 19, no. 5, pp. 1264-1274, Sept.-Oct. 1989, doi: 10.1109/21.44046. keywords: {Humans;Image texture analysis;Pixel;Information analysis;Layout;Computational efficiency;Anthropometry;Visual perception;Surface texture;Digital images},

5. Sun, Chengjun and William G. Wee. “Neighboring gray level dependence matrix for texture classification.” Comput. Graph. Image Process. 1982; 20: 297.

6. CHU, A.; SEHGAL, Chandra M.; GREENLEAF, James F. Use of gray value distribution of run lengths for texture analysis. Pattern recognition letters. 1990;11: 415-419.

7. Lambin, Philippe, et al. "Radiomics: extracting more information from medical images using advanced feature analysis." European journal of cancer 2012; 48: 441-446.

8. Xu, J., Faruque, J., Beaulieu, C.F. et al. A Comprehensive Descriptor of Shape: Method and Application to Content-Based Retrieval of Similar Appearing Lesions in Medical Images. J Digit Imaging. 2012;25:121–128. https://doi.org/10.1007/s10278-011-9388-8.

9. T. Ojala, M. Pietikainen and T. Maenpaa, "Multiresolution gray-scale and rotation invariant texture classification with local binary patterns," in IEEE Transactions on Pattern Analysis and Machine Intelligence. 2002;24:971-987. doi: 10.1109/TPAMI.2002.1017623.

10. Gabor, Dennis. “Theory of communication.” Journal of the Institution of Electrical Engineers - Part I: General. 1946;94: 58-58.

**Table S1: Radiomics features**

| FIRSTORDER | 10Percentile, 90Percentile, Energy, Entropy, InterquartileRange, Kurtosis, Maximum, MeanAbsoluteDeviation, Mean, Median, Minimum, Range, RobustMeanAbsoluteDeviation, RootMeanSquared, Skewness, StandardDeviation, TotalEnergy, Uniformity, Variance |
| --- | --- |
| SHAPE | Compactness, Elongation, Flatness, LeastAxisLength, MajorAxisLength, Maximum2DDiameterColumn, Maximum2DDiameterRow, Maximum2DDiameterSlice, Maximum3DDiameter, MeshVolume, MinorAxisLength, SphericalDisproportion, Sphericity, SurfaceArea, SurfaceVolumeRatio, VoxelVolume, Width, Height, Depth, Radial distance, roughness, convexity, solidity, area, Eccentricity |
| GLCM | Autocorrelation, ClusterProminence, ClusterShade, ClusterTendency, Contrast, Correlation, DifferenceAverage, DifferenceEntropy, DifferenceVariance, Id, Idm, Idmn, Idn, Imc1, Imc2, InverseVariance, JointAverage, JointEnergy, JointEntropy, MCC, MaximumProbability, SumAverage, SumEntropy, SumSquares |
| GLSZM | GrayLevelNonUniformity, GrayLevelNonUniformityNormalized, GrayLevelVariance, HighGrayLevelZoneEmphasis, LargeAreaEmphasis, LargeAreaHighGrayLevelEmphasis, LargeAreaLowGrayLevelEmphasis, LowGrayLevelZoneEmphasis, SizeZoneNonUniformity, SizeZoneNonUniformityNormalized, SmallAreaEmphasis, SmallAreaHighGrayLevelEmphasis, SmallAreaLowGrayLevelEmphasis, ZoneEntropy, ZonePercentage, ZoneVariance |
| GLRLM | GrayLevelNonUniformity, GrayLevelNonUniformityNormalized, GrayLevelVariance, HighGrayLevelRunEmphasis, LongRunEmphasis, LongRunHighGrayLevelEmphasis, LongRunLowGrayLevelEmphasis, LowGrayLevelRunEmphasis, RunEntropy, RunLengthNonUniformity, RunLengthNonUniformityNormalized, RunPercentage, RunVariance, ShortRunEmphasis, ShortRunHighGrayLevelEmphasis, ShortRunLowGrayLevelEmphasis |
| NGTDM | Busyness, Coarseness, Complexity, Contrast, Strength |
| GLDM | DependenceEntropy, DependenceNonUniformity, DependenceNonUniformityNormalized, DependenceVariance, GrayLevelNonUniformity, GrayLevelVariance, HighGrayLevelEmphasis, LargeDependenceEmphasis, LargeDependenceHighGrayLevelEmphasis, LargeDependenceLowGrayLevelEmphasis, LowGrayLevelEmphasis, SmallDependenceEmphasis, SmallDependenceHighGrayLevelEmphasis, SmallDependenceLowGrayLevelEmphasis |
| GABOR | FeatureDescriptorMean |
| GABOR | FeatureDescriptorStandardDeviation |
| LBP | FeatureDescriptorHisogram |
